# The long weight: association between distressed communities index and long-term weight outcomes following bariatric surgery

**DOI:** 10.1007/s00464-023-10158-y

**Published:** 2023-06-27

**Authors:** Alexandra J. Johns, M. Siobhan Luce, Mason J. Kaneski, Ryan A. Lowery, Barbara Jachniewicz, Angela Salas, Randi McCreary, Raquel M. Russell, Victoria Lyo, Mohammed R. Ali, Shushmita M. Ahmed

**Affiliations:** 1 Department of Surgery, University of California, Davis, 2335 Stockton Blvd, 6thFloor, Sacramento, CA 95817 USA; 2 Division of Foregut, Metabolic, and General Surgery, University of California, Davis, Sacramento, USA; 3 Center for Metabolic and Alimentary Science, University of California, Davis, Sacramento, USA; 4 University of California, Davis Health, Sacramento, USA

**Keywords:** Weight recurrence, Socioeconomic status, Bariatric surgery, Long-term weight loss

## Abstract

**Background:**

Socioeconomic status (SES) is multifactorial, and its effect on post-bariatric weight recurrence is unclear. Distressed Community Index (DCI) is a composite SES score measuring community economic well-being. This study aims to evaluate the effect of DCI on long-term post-bariatric weight outcomes.

**Methods:**

Retrospective analysis of patients undergoing primary laparoscopic Roux-en-Y gastric bypass or sleeve gastrectomy between 2015 and 2020 was performed. All weights in the electronic medical record (EMR), including non-bariatric visits, were captured. Patients were stratified into low tier (LT) and high tier (HT) DCI groups.

**Results:**

Of 583 patients, 431 (73.9%) were HT and 152 (26.1%) were LT. Average bariatric follow up was 1.78 ± 1.6 years and average postoperative weight in the EMR was 3.96 ± 2.26 years. Rates of bariatric follow up within the last year were similar (13.8% LT vs 16.2% HT, *p* = 0.47). LT had higher percent total body weight loss (%TWL; 26% LT vs 23% HT, *p* < 0.01) and percent excess weight loss (%EWL; 62% vs 57%, *p* = 0.04) at 1 year on univariate analysis. On multivariate linear regression adjusting for baseline characteristics and surgery type, there were no differences in %EWL between groups at 1 year (*p* = 0.22), ≥ 3 years (*p* = 0.53) or ≥ 5 years (*p* = 0.34) postop. While on univariate analysis LT only trended towards greater percentage of patients with > 15% increase from their 1-year weight (33.3% LT vs 21.0% HT, *p* = 0.06), on multivariate analysis this difference was significant (OR 2.0, LT 95%CI 1.41–2.84). There were no differences in the percentage of patients with > 15% decrease in %EWL from 1 to 3 + years postop between groups (OR 0.98, LT 95% CI 0.72–1.35).

**Conclusions:**

While low tier patients had similar weight loss at 1 year, they were twice as likely to have weight recurrence at ≥ 3 years. Further studies are needed to identify factors contributing to greater weight recurrence among this population.

In the face of the obesity pandemic, bariatric surgery is the most effective and durable treatment for obesity and its associated comorbidities [1, 2]. Nonetheless, weight recurrence is a known phenomenon postoperatively. The true prevalence of weight recurrence is unclear and varies widely in the literature, from 9 to 91%, largely due to a lack of any standardization in definition or reporting [3–6].

Several studies have identified age, sex, and initial body mass index (BMI) as risk factors for weight recurrence following bariatric surgery [7–9]. Socioeconomic status (SES) has also been identified as a risk factor for poor long-term post-bariatric outcomes; however, its association with long-term weight outcomes remains mixed [10–15]*.* SES is multifactorial, making it difficult to accurately calculate. As with weight recurrence, differing definitions have been used for SES, and individual variables (e.g., insurance type, household income, etc.) are often used to represent this complex measure. A Swedish national study found immigration status and proximity to large cities to be risk factors for lower 5-year percent excess body mass index loss (%EBMIL) after Roux-en-Y gastric bypass (RYGB) [16]. Similarly, Carden et al. showed greater 10-year post-RYGB %EBMIL among higher income patients [14]. In contrast, on multivariate analysis, both Hecht et al. and Kitamura et al. showed that income was not significantly associated with weight outcomes [10, 17]. Indeed, the oversimplified and variable definitions of SES in the literature likely contributes to the inconsistent relationship seen between SES and sustained weight loss following bariatric surgery.

The Distressed Communities Index (DCI) was developed by the Economic Innovation Group to provide a more comprehensive assessment of SES through a measure of community economic well-being. DCI is a geocoded composite score that takes into account seven SES factors not found in the medical record: community education rates, poverty rate, income, housing status, employment status, rate of change in employment, and business growth [18]. DCI has been previously used in surgical literature to aid in risk stratification and predict outcomes [19]. For instance, compared to patients from prosperous communities, patients from distressed communities have higher mortality rates after cardiac surgery and limb loss after vascular bypass surgery [20, 21].

Few studies have evaluated the effect of DCI on bariatric outcomes, including weight loss, and no studies have looked at its association with long-term weight recurrence [22]. Thus, given its utility as a more robust SES measure, we aimed to determine the effect of DCI status on long-term weight outcomes and weight recurrence following bariatric surgery at our institution.

## Methods

A retrospective analysis of all patients over 18 years of age undergoing primary laparoscopic Roux-en-Y gastric bypass (RYGB) or sleeve gastrectomy (SG) between 2015 and 2020 was performed at a single academic institution. The study was approved by the intuitional review board at the University of California, Davis. Clinical data were extracted from the electronic medical record (EMR) and included demographics, preoperative characteristics, wait time to surgery from initial consultation, and postoperative follow up and weights. All weights within the health care system, including bariatric and non-bariatric visits, as well as in person and telehealth visits, were captured. Patients undergoing surgery after March 2020 were eliminated to reduce any confounding effects from the COVID-19 pandemic.

### Distressed communities index

DCI defines community borders by zip code and assigns a score from 0 (no distress) to 100 (severe distress). Communities are then ranked into 5 categories: distressed, at risk, mid-tier, comfortable, and prosperous [18]. Zip codes were obtained from the EMR to score and categorize each patient into their respective DCI community. Patients were then stratified into high tier DCI status (HT), which includes mid-tier, comfortable and prosperous communities, and low tier DCI status (LT) which includes at-risk and distressed communities.

### Weight calculations

Postoperative weight loss was reported in standardized bariatric measures: percent total body weight loss (%TWL) and percent excess weight loss (%EWL) [23]. Excess weight was calculated as the difference between initial weight and ideal body weight, with ideal body weight calculated based on a BMI of 25 kg/m^2^. Weight loss was recorded at 1 year postop, 3–5 years postop, and > 5 years postop. As there is currently no standardized definition for weight recurrence, two previously used measurements were adapted for this patient population: > 15% increase from nadir postoperative weight and > 15% decrease from nadir %EWL [3, 4, 24, 25]. Although both of these calculations have been used in prior research to define weight recurrence, they represent different levels of weight regain as 15% decrease from nadir %EWL is often a much lower amount of weight regain than > 15% increase from nadir postoperative weight. These two definitions were chosen to analyze both a mild weight recurrence (15% decrease from nadir %EWL) and a larger weight recurrence (15% increase from nadir weight). To standardize the calculations, we defined the nadir to be at 1 year following bariatric surgery. Weight recurrence was calculated based on weight change from 1-year postop to ≥ 3 postop.

Calculations for weight outcomes and recurrence are shown below:

$$\begin{array}{*{20}c} {\% {\text{TWL }} = \, \left[ {\left( {{\text{initial weight}} - {\text{current weight}}} \right)/{\text{initial weight}}} \right] \times {1}00} \\ {\% {\text{EWL }} = \, \left[ {\left( {{\text{initial weight}} - {\text{current weight}}} \right)/{\text{excess weight}}} \right] \times {1}00} \\ {{\text{where}}:{\text{ Excess Weight }} = \, \left( {{\text{initial weight }}{-}{\text{ ideal body weight}}} \right){\text{ and ideal body weight is calculated from BMI 25 kg}}/{\text{m}}^{{2}} } \\ {{\text{Weight recurrence if }} > {15}\% {\text{ increase from 1}} - {\text{year postoperative weight}}} \\ {{\text{where }}\% {\text{ increase }} = \, \left[ {\left( {{\text{recent weight}} - {\text{1 year weight}}} \right)/{\text{1 year weight}}} \right] \times {1}00} \\ {{\text{OR}}} \\ {{\text{Weight recurrence if }} > {15}\% {\text{ decrease from 1}} - {\text{year postoperative }}\% {\text{EWL}}} \\ {{\text{where }}\% {\text{ decrease }} = \left[ {{1} - \left( {{\text{Current }}\% {\text{EWL}}/{\text{1 year }}\% {\text{EWL}}} \right)} \right] \times {1}00} \\ \end{array}$$

Statistical analysis was done using R Studio Software, version 4.1.2 (R Studio Team, 2020, PBC, Boston, MA) by the primary author (AJJ). Student’s *t*-test were used to compare continuous variables and chi-squared analyses were used to compare categorical variables between high and low tier groups. Multivariate regressions were performed controlling for age, sex, race, type of surgery, preop BMI, American Society of Anesthesiologists (ASA) status, and insurance type.

## Results

Of 582 patients undergoing primary RYGB and SG, 431 (73.9%) were in the HT group and 152 (26.1%) were in the LT group. Demographic data are summarized in Table 1. Overall, mean age was 48.7 ± 12.3 years, and 76.4% of patients were women. There were no differences between LT and HT in age, sex, race, or ethnicity. However, LT patients were more likely to have public insurance than HT patients (32.0% HT vs 48.7% LT, *p* < 0.01).

**Table 1:** Demographic data of all patients undergoing primary bariatric surgery

	Overall	High tier DCI	Low tier DCI	*p*-value
	*n* = 583	*n* = 431 (73.9%)	*n* = 152 (26.1%)	
Participant characteristics				
Patient age in years, mean (*SD*)	48.7 (± 12.3)	48.9 (± 12.4)	47.7 (± 12.1)	0.291
Min. Age	18	18	22	
Max. Age	80	80	72	
Female %	76.4%	74.2%	82.2%	0.118
DCI Breakdown *n* (%)				
5	165 (28.3%)	165 (38.3%)	–	
4	151 (25.9%)	151 (35.0%)	–	
3	115 (19.7%)	115 (26.7%)	–	
2	125 (21.4%)	–	125 (82.2%)	
1	27 (4.6%)	–	27 (17.8%)	
NA	5 (0.9%)	–	–	
Race breakdown *n* (%)				0.156
White	414 (71%)	311 (72.2%)	98 (64.5%)	
African American	66 (11.3%)	42 (9.7%)	24 (15.8%)	
Hispanic	65 (11.1%)	43 (10.0%)	22 (14.5%)	
Native Hawaiian or Pacific Islander	8 (1.4%)	6 (1.4%)	2 (1.3%)	
Asian	8 (1.4%)	6 (1.4%)	2 (1.3%)	
American Indian or Alaska Native	7 (1.2%)	5 (1.2%)	2 (1.3%)	
Other or undetermined	20 (3.4%)	18 (4.2%)	2 (1.3%)	
% Hispanic ethnicity	17.5%	15.8%	22.4%	0.135
Insurance breakdown *n* (%)				< 0.001*
All private	373 (64.0%)	293 (68.0%)	78 (51.3%)	
All public	215 (36.9%)	138 (32.0%)	74 (48.7%)	
Further breakdown of public insurance				
Medicare	154 (71.6%)	102 (73.9%)	50 (67.6%)	
Medicaid	52 (24.2%)	30 (21.7%)	21 (28.4%)	
Other (Tricare, VA, etc.)	8 (3.7%)	6 (4.3%)	2 (2.7%)	

Statistics comparing High Tier DCI to Low Tier DCI groups

*SD* standard deviation

*p < 0.05

Preoperative characteristics and comorbidities are summarized in Table 2. While there were no differences between HT and LT groups in BMI at surgery (44.0 ± 7.3 kg/m2 HT vs 44.9 ± 7.8 kg/m2 LT, *p* = 0.25), percent weight loss prior to surgery (3.19 ± 15.9% HT vs 3.66 ± 7.4% LT, *p* = 0.63), or number of days from initial consult to surgery (238.7 ± 362.8 days HT vs 251.1 ± 390.4 days LT, *p* = 0.73), LT patients were more likely to have sleep apnea (66.4% HT vs 77.6% LT, *p* < 0.01) and non-alcoholic fatty liver disease (7.4% HT vs 15.1%LT, *p* = 0.02). Overall health as measured by ASA status was similar between both groups (*p* = 0.48). Finally, a greater percentage of LT patients underwent RYGB (54.3% HT vs 63.2% LT, *p* = 0.07), but this difference was not statistically significant.

**Table 2:** Preoperative characteristics and surgery type

	Overall	High tier DCI	Low tier DCI	*p*-value
Pre-operative characteristics				
Initial weight, Kg				
Mean (SD)	130.4 (± 30.9)	130.2 (± 31.3)	130.9 (± 29.9)	0.805
Excess weight, Kg				
Mean (SD)	54.2 (± 21.6)	53.8 (± 21.6)	55.4 (± 21.9)	0.446
Weight lost pre-op, Kg				
Mean (SD)	5.75 (± 15.3)	5.76 (± 15.9)	5.76 (± 13.8)	0.998
Weight lost pre-op, % total weight loss^a^				
Mean (SD)	3.31% (± 14.1)	3.19% (± 15.9)	3.66% (± 7.4)	0.626
BMI at surgery				
Mean (SD)	44.3 (± 7.4)	44.0 (± 7.3)	44.9 (± 7.8)	0.252
Min. BMI	32.1	32.1	33.2	
Max. BMI	79.9	75.0	79.9	
Days to surgery from initial consult				
Mean (SD)	241.9 (± 368.5)	238.7 (± 362.8)	251.1 (± 390.4)	0.731
Min. days	19	19	38	
Max. days	3370	3370	2523	
Comorbidities				
Hypertension	359 (61.6%)	268 (62.2%)	88 (57.9%)	0.357
Diabetes	218 (37.4%)	157 (36.4%)	58 (38.2%)	0.706
Sleep apnea	408 (70.0%)	286 (66.4%)	118 (77.6%)	0.006*
Cardiac disease	70 (12.0%)	48 (11.1%)	22 (14.5%)	0.304
Chronic obstructive pulmonary disease	11 (1.9%)	9 (2.1%)	2 (1.3%)	0.504
Liver disease	55 (9.4%)	32 (7.4%)	23 (15.1%)	0.016*
Psychiatric disease	296 (50.8%)	208 (48.3%)	84 (55.3%)	0.138
Peripheral vascular disease	19 (3.3%)	13 (3.0%)	6 (3.9%)	0.603
ASA status *n* (%)				0.476
ASA-2	73 (12.5%)	50 (11.6%)	23 (15.1%)	
ASA-3	509 (87.3%)	377 (87.5%)	127 (83.6%)	
ASA-4	6 (1.0%)	4 (0.9%)	2 (1.3%)	
Type of surgery *n* (%)				0.072
Roux-En-Y bypass	331 (56.8%)	234 (54.3%)	96 (63.2%)	
Sleeve gastrectomy	257 (44.1%)	197 (45.7%)	56 (36.8%)	

Statistics comparing High Tier DCI to Low Tier DCI groups

*SD* standard deviation

*p < 0.05

^a^From initial weight

Overall, 63.3% of patients (*n* = 369) attended their 1-year bariatric follow-up visit, and 15.8% of patients followed up in bariatric clinic within the past 12 months. Rates of documented weights (bariatric and non-bariatric) at 3–5 years and > 5 years are shown in Table 3. There were no differences in follow up rates between DCI groups (Table 3). Additionally, HT and LT groups had similar duration of follow up in bariatric clinic (20.8 ± 19.4 months vs 23.0 ± 19.7 months, *p* = 0.47). When including non-bariatric visits, patients’ last recorded weight was on average 3.96 ± 2.26 years postop (*p* = 0.94).

**Table 3:** Short and long-term follow up rates and weight outcomes

	Overall	High tier DCI	Low tier DCI	*p*-value
Follow up				
Attended 1-year bariatric visit, *n* (%)	369 (63.3%)	273 (63.3%)	95 (62.5%)	0.854
Attended bariatric visit in past 12 months, *n* (%)	92 (15.8%)	70 (16.2%)	21 (13.8%)	0.466
Months of follow-up with bariatric clinic				
Mean (SD)	21.3 (± 19.4)	20.8 (± 19.4)	23.0 (± 19.7)	0.237
Rate of weight documented for given time point				0.343
3–5 years, *n* (%)	145 (24.9%)	113 (26.2%)	31 (20.4%)	
5 + years, *n* (%)	231 (39.6%)	168 (39.0%)	62 (40.8%)	
Short-term weight loss				
% TWL at 1-year				
Mean (SD)	23.9% (± 9.09)	23.2% (± 8.99)	26.0% (± 9.03)	0.009*
% EWL at 1-year				
Mean (SD)	0.58 (± 0.23)	0.57 (± 0.23)	0.62 (± 0.22)	0.041*
Quartile of % Total Weight Lost at 1 year				0.058
< 10%	5.9%	6.2%	4.2%	
10–25%	49.9%	53.1%	41.1%	
> 25%	44.2%	40.7%	54.7%	
Quartile of Excess Weight Lost at 1 year				0.111
< 30%	10.8%	11.7%	7.4%	
30–60%	44.4%	46.5%	38.9%	
> 60%	44.7%	41.8%	53.7%	
Long-term Weight Loss				
%TWL at 3–5 years postop				
Mean (SD)	17.2% (+ -11.8)	16.8% (± 12.2)	18.5% (± 10.2)	0.453
%EWL at 3–5 years postop				
Mean (SD)	0.44 (± 0.32)	0.43 (± 0.33)	0.45 (± 0.26)	0.720
%TWL at 5 + years postop				
Mean (SD)	16.3% (± 11.6)	15.5% (± 11.9)	18.5% (± 10.6)	0.069
%EWL at 5 + years postop				
Mean (SD)	0.40 (± 0.30)	0.38 (± 0.30)	0.46 (± 0.27)	0.073
Quartile of % total weight lost at 3 + years (%)				0.166
< 10%	26.7%	29.2%	19.4%	
10–25%	51.1%	49.8%	54.8%	
> 25%	22.2%	21.0%	25.8%	
Quartile of excess weight lost at 3 + years (%)				0.430
< 30%	35.6%	37.3%	30.1%	
30–60%	36.6%	35.9%	38.7%	
> 60%	27.8%	26.7%	31.2%	
Weight recurrence^a,b^				
> 15% Increase from 1-year weight, n (%)	64 (24.1%)	42 (21.0%)	22 (33.3%)	0.062
> 15% Decrease from 1-year % EWL, n (%)	164 (61.7%)	126 (63.0%)	38 (57.6%)	0.441

Statistics comparing High Tier DCI to Low Tier DCI groups

*SD* standard deviation

*p < 0.05

^a^Evaluating weight change from 1 year to 3 + year postop

^b^Sample size limited to patients with weights documented for both 1-year and 3 + years postoperatively; HT has *n* = 200 patients and LT has *n* = 66 patients

On univariate analysis, LT patients had significantly greater %TWL (23.2 ± 8.99% HT vs 26.0 ± 9.03% LT, *p* = 0.01) and %EWL (57% vs 62%, *p* = 0.04) at 1-year postop. While there were no differences in weight loss between groups at 3 to 5 years, LT patients exhibited greater %TWL (15.5 ± 11.9% HT vs 18.5 ± 10.6% LT, *p* = 0.06) and %EWL (38 ± 30.0% vs 46 ± 27.0%, *p* = 0.07) beyond 5 years, but this did not reach statistical significance. However, on multivariable linear regressions, controlling for preoperative characteristics and type of surgery, these differences were no longer seen (Table 4).

**Table 4:** Multivariate linear regression, adjusting for baseline characteristics and type of surgery

	% Total weight loss		% Excess weight loss	
	Slope for LT	*p*-value	Slope for LT	*p*-value
Percent lost at 12 Months	1.27	0.19	0.03	0.22
Percent lost at ≥ 3 Years	0.88	0.52	2.22	0.53
Percent lost at ≥ 5 Years	1.51	0.39	4.25	0.34

Control variables: Age, Sex, Race, Type of Surgery, Preop BMI, ASA, Insurance Type

In this population, 376 patients (64.5%) had weights documented for ≥ 3 years postoperatively and 231 (39.6%) had weights documented for ≥ 5 years postoperatively, with similar distributions of HT and LT groups (Table 3). Given our sample size, midterm and long-term weights were grouped into ≥ 3 postop weight category to measure weight recurrence. On univariate analysis, a greater percentage of LT patients were found have > 15% increase from their 1-year postop weight, although this difference did not reach statistical significance (21.1% HT vs 33.3% LT, *p* = 0.06). However, on multivariate logistic regression, LT patients were twice as likely to have > 15% weight increase from their 1-year postop weight (OR 2.0, LT 95% CI 1.41–2.84). On both univariate and multivariate regression, there were no differences between groups in the percentage of patients with > 15% decrease from 1-year %EWL (Table 5). Multivariate regression also confirmed no differences between groups in attendance of 1-year bariatric visit or bariatric visit within the last 12 months.

**Table 5:** Multivariable logistic regressions adjusting for baseline characteristics and type of surgery

	Odd’s ratio for low tier DCI status	95% CI
Attended 1-year bariatric visit	0.95	0.77–1.17
Attended bariatric visit in past 12 month	0.69*	0.52–0.91
> 15% Increase from 1-year weight^a,b^	2.00*	1.41–2.84
> 15% Decrease from 1-year % EWL^a,b^	1.06	0.78–1.45

Control variables: Age, Sex, Race, Type of surgery, Pre-Op BMI, ASA, Insurance type

*Statistically significant (95% CI does not cross 1)

^a^Evaluating weight change from 1 year to 3 + year postop

^b^Sample size limited to patients with weights documented for both 1-year and 3 + years postoperatively; HT with *n* = 200 patients and LT with n = 66 patients

## Discussion

Obesity is a lifelong chronic disease that can be managed but not cured, therefore the durability of therapies, including bariatric surgery, must be optimized. It is crucial to understand the risk factors that affect weight outcomes, especially since the magnitude and maintenance of weight loss significantly impacts improvement in metabolic health and obesity-associated comorbidities. SES has been implicated as one such risk factor, but measures of SES and its effect on weight loss have been variable [5, 7, 9, 10]. Given its role as a comprehensive measure for SES, we used DCI as a means to better understand the impact of SES on weight loss and weight recurrence after bariatric surgery.

In this study, LT patients were similar to HT patients in demographics and preoperative profiles. However, LT patients were more likely to have public insurance and undergo RYGB. Univariate analysis showed greater 1-year weight loss among LT patients; however, this was not statistically significant on multivariate analysis, consistent with other studies which showed SES did not affect short-term weight outcomes [7, 11, 17]. This finding is encouraging as it indicates that, despite poorer resources, LT patients can have the same short-term weight loss benefits as their HT counterparts. The difference seen on univariate analysis may be due, in part, to the higher prevalence of RYGB among the LT population. While not statistically significant at *p* = 0.07, this difference is clinically significant, as RYGB has been shown to provide greater short and long-term weight loss [26–28]. Thus, upon controlling for surgery type, the weight loss difference initially seen no longer exists.

Univariate analysis also showed greater weight loss maintenance at > 5 years among LT that was not statistically significant. Once again, this difference was no longer seen on multivariate regression, consistent with previous studies evaluating the role of SES on long-term outcomes [10, 22, 29]. In 2021, Pouchucq et al. showed no association between the European Deprivation Index and 12-year postoperative %TWL. Similarly, Mehaffey et al. found no differences in 10-year weight loss using continuous DCI scores (0–99).

Interestingly, we found that, although weight loss was similar between groups across all times points, LT DCI status was an independent predictor for weight recurrence after controlling for preoperative characteristics and surgery type. This was only observed for weight recurrence based on total body weight but not for change in %EWL. This has several implications. First, while absolute weight loss was similar between HT and LT, there may be a difference in the rate of post-bariatric weight recurrence between 1-year and ≥ 3 years postoperatively among the two groups. Second, the percent difference from 1-year weight versus 1-year %EWL may represent different degrees of weight recurrence [30, 31]. For example, 33% of LT patients had > 15% gain from 1-year weight, but 58% of LT patients exhibited > 15% decrease from 1-year %EWL. This implies that the patients who demonstrated significant change in total body weight could represent a subset of the population who had change from 1-year %EWL. In other words, change from % EWL may represent mild weight recurrence while change from 1-year weight may represent moderate recurrence. So, while there may not be a difference in mild recurrence between the groups, LT patients were more susceptible to moderate recurrence following surgery. Longer-term studies with larger sample sizes are needed to better evaluate differences in the rate of weight recurrence among HT vs LT and whether differences seen in our study persistent beyond 10 years.

The difference in statistical significance based on weight recurrence definition highlights the need for an established standard for reporting weight recurrence [30]. Like our study, prior groups have found different results when using differing calculations even amongst the same patient population [3, 30–32]. Moreover, there is no defined guideline for the degree of weight gain that is acceptable or expected after surgery (and, thus, not considered weight recurrence) [4]. Ultimately, this study adds to the body of literature demonstrating the importance for a universally accepted definition of weight recurrence that will allow for more consistent research to help identify and modify predictors for weight recurrence as is the goal of the ASMBS POWER task force [4].

### Limitations

There were several limitations to this study including its retrospective nature. Our cohort saw a moderate attrition rate of long-term follow up. This is a common challenge faced by longitudinal studies. In a systematic review of 7173 studies on bariatric outcomes, less than 3% had 80% follow up rates [33]. Studies show progressively poorer follow up rates with time, as low as 30% at 2 years, and < 10% follow up at 10 years [22, 32, 34, 35]. To address the attrition rate, we augmented our sample size by including non-bariatric visit weights. This increased our cohort size to 376 patients (64.5% of the population) with ≥ 3 years of postoperative weight. Moreover, follow up was similar for HT and LT groups at all times, thereby minimizing further confounders. Also, the lack of a standardized definition blurs the lines between expected weight regain and clinically significant weight recurrence. We tried to mitigate this by using previously reported criteria. Nonetheless, weight recurrence has significant long-term clinical implications and without an established definition, potential risk factors may go unrecognized. The ASMBS POWER task force will ideally reconcile this issue with universally accepted definitions.

In addition, the Distressed Communities Index analyzes communities and has information on communities nationwide, however, this study was done using patients from a single institution in Northern California. While the patient population includes rural, suburban, and urban patients, this study cannot be generalized to all cities or countries as the effects of community economic well-being may differ in different regions. A multi-institutional study using longitudinal data including long-term follow-up and repeated weight measurements would allow for more generalizable results. Lastly, this study combines outcomes from both Roux-en-Y gastric bypass and sleeve gastrectomy which can lead to different weight outcomes as mentioned in the discussion section above. Future studies could isolate single procedures and compare community well-being effects specific to that procedure.

## Conclusion

Ours is the first study to evaluate the effect of DCI on weight recurrence following bariatric surgery. Low tier patients were found to be 2 times more likely to have weight recurrence at ≥ 3 years postop. While the similarities in short and long-term weight outcomes are reassuring, the higher rates of weight recurrence seen among low tier patients indicates that this population is at greater risk long-term. This study highlights both the need for further investigation of SES on long-term weight recurrence and the need for a well-accepted calculation of weight recurrence. Future studies will evaluate longer outcomes including 10 and 20-year weight recurrence as bariatric surgery is intended to be a durable weight loss treatment option.
